# Supplementary material for: Six months survival and risk factors for attrition for patients detected with cryptococcal antigenemia through screening in Malawi
Source: PLoS One. 2023 May 4;18(5):e0284367. doi: 10.1371/journal.pone.0284367 (PMC10159159; doi:10.1371/journal.pone.0284367)
Supplement: S1 File — (DOCX) [file pone.0284367.s002.docx]

**Supplementary Table 1: Treatment prescribed by diagnosis**

| **Diagnosis** | **CrAg-positive** | **No treatment prescribed** | **Fluconazole^b^** | **Referred to another location^c^** | **Amphotericin B only** | **Amphotericin B + fluconazole** | **Unknown** |
| --- | --- | --- | --- | --- | --- | --- | --- |
| **Asymptomatic** | 49 (43.8) | 0 (-) | 49 (100) | - | 0 (-) | 0 (-) | 0 (-) |
| **Symptomatic CSF-negative (or no LP)** | 26 (23.2) | 1 (3.8) | 25 (96.2) | - | 0 (-) | 0 (-) | 0 (-) |
| **CSF-positive** | 33(29.5) | 0 (-) | 6 (18.2) | 18 (54.5) | 1 (3.0) | 18 (54.5) | 8 (24.2) |
| **Missing symptom status** | 4 (3.6) | 0 (-) | 0 (-) | - | 0 (-) | 0 (-) | 4 (100) |
| **Totals** | **112** | **1 (0.9)** | **80 (71.4)** | **18 (16.1)** | **1 (0.9)** | **18 (16.1)** | **12 (10.7)** |

Note: ^a^ This patient was admitted and died the day after CrAg test prior to receiving a fluconazole prescription ^b^ includes 1 patient who was prescribed a sub-optimal dose of fluconazole pre-emptive therapy; ^c^ does not include CM patients at Mzuzu Central Hospital, as amphotericin B was available onsite

**Results:** Table 1 displays the prescriptions that were recorded on the clinic charts for CrAg positive patients. Coverage of fluconazole prescribing was very high for asymptomatic and symptomatic/CSF-negative patients. Prescribing of amphotericin B and fluconazole for patients diagnosed with CM was 54.5%. Of the 6 CM patients who were recorded as being prescribed fluconazole only, clinical notes indicated that 2 of these (both from Thyolo DH) were due to amphotericin B being out of stock at QECH during one period of the study. Patients diagnosed with CM at Thyolo, Bangwe, or Dedza were referred to non-pilot sites for Amphotericin B and treatment info was not always communicated back to the referring facility. Therefore, there is considerable missingness in the prescription info for CM patients.

**Supplementary Table 2: Clinical information on the 4 patients who developed symptoms following asymptomatic presentation or resolution of baseline symptoms**

| **Patient** | **Status at screening** | **Reason for Enrollment** | **Treatment prescribed** | **Days until symptoms** | **Total days in follow up** | **Notes** |
| --- | --- | --- | --- | --- | --- | --- |
| **A** | Asymptomatic | Treatment failure | Fluconazole | 14 | 180 | Headache reported at first follow up visit. Subsequently resolved |
| **B** | Asymptomatic | Inpatient | Fluconazole | 8 | 8 | Reported headache and fever on the 18^th^; reported to have died of TB and severe bacterial infections |
| **C** | Symptomatic with headache and fever; CSF-negative | Treatment failure | Fluconazole | 76 | 180 | Reported headache and fever on January 9^th^; no CSF test done; symptoms subsequently resolved |
| **D** | Asymptomatic | Treatment failure | Fluconazole | 46 | 180 | Reported headache at second follow visit; CSF test not done; symptoms subsequently resolved |
